# Supplementary material for: Chemotherapeutic Strategies with Valnemulin, Tilmicosin, and Tulathromycin to Control Mycoplasma hyopneumoniae Infection in Pigs
Source: Antibiotics (Basel). 2022 Jul 4;11(7):893. doi: 10.3390/antibiotics11070893 (PMC9311983; doi:10.3390/antibiotics11070893)
Supplement: Supplementary file 1 [file antibiotics-11-00893-s001.zip › antibiotics-1728070-supplementary.pdf]

Table S1. Medians of *M. hyopneumoniae* quantifications in the nasal swab among groups over time for each time point (T) from T1 to T6 (151 days of age for G1, G2, and G3 and at 145 days of age for G4).

Points	Group	Median	Median standard error	P value
T1 (24 days)	G1	0.00[a]	3.08	2.7×10^{-1}
	G2	0.00[a]	0.00	
	G3	0.00[a]	16.5	
	G4	0.00[a]	31.5	
T2 (45 days)	G1	0.00[a]	0.00	1.4×10^{-1}
	G2	0.00[a]	11.6	
	G3	0.00[a]	0.00	
	G4	0.00[a]	1.61	
T3 (63 days)	G1	0.00	0.00	NA
	G2	0.00	0.00	
	G3	0.00	0.00	
	G4	0.00	0.00	
T4 (93 days)	G1	0.00[a]	0.00	5.5×10^{-1}
	G2	0.00[a]	0.04	
	G3	0.00[a]	5.53	
	G4	0.00[a]	67.6	
T5 (123 days)	G1	0.00[a]	25.6	1.3×10^{-1}
	G2	0.00[a]	23.8	
	G3	0.00[a]	86.1	
	G4	0.00[a]	23.3	
T6 (151 days)	G1	0.00[a]	0.00	7×10^{-2}
	G2	0.00[a]	73.9	
	G3	0.00[a]	0.00	
	G4	0.00[a]	0.00	

* Equal letters indicate no significant differences by the non-parametric Kruskal - Wallis test ($P > 0.05$).
** As no detections were recorded in the period, it was not possible to obtain median values and standard error, nor to perform a comparison test among the groups.

Table S2. Affected lung area determined by macroscopic evaluation.

**Means of pulmonary consolidation area**			
**Group**	**Mean**	**Standard error**	**P value**
G1	9.82[a]	2.02	6×10^{-1}
G2	6.98[a]	1.31	
G3	7.47[a]	1.57	
G4	7.69[a]	1.27	

* Equal letters indicate no significant differences by the non-parametric Kruskal - Wallis test ($P > 0.05$).

Table S3. Median degree of injury of the lungs collected in the slaughterhouse in the histopathological examination.

Median values for degrees of injury			
**Group**	**Median**	**Median Standard Error**	**P value**
G1	3.00[a]	2.60	0.13
G2	3.00[a]	2.13	
G3	2.50[a]	1.71	
G4	3.00[a]	2.83	

* Equal letters indicate that the *medians* do not differ at the level of 0.05 by the nonparametric Kruskal - Wallis test.

Table S4. IgG anti-*M. hyopneumoniae* S/P values individualized by animal over time (T1 to T6).

		IgG anti – *Mhyo* S/P values					
**Groups**	**ID**	**T1 (24 days)**	**T2 (45 days)**	**T3 (63 days)**	**T4 (93 days)**	**T5 (123 days)**	**T6 (151 days)**
G1	283	0.539	0.253	0.058	0.284	0.602	2.018
G1	287	0.485	0.194	0.202	0.799	1.959	2.143
G1	291	0.161	-0.008	0.074	-0.008	0.008	0.536
G1	298	0.001	0.077	0.454	0.447	0.525	-0.082
G1	301	1.789	0.093	0.081	0.178	1.343	1.680
G1	303	0.293	0.058	0.310	1.855	1.727	1.761
G1	310	0.010	-0.025	0.148	0.011	-0.034	0.577
G1	319	-0.036	-0.010	0.470	0.437	1.645	1.586
G1	331	0.147	-0.003	0.055	1.522	1.756	1.463
G1	338	0.199	0.029	0.249	0.308	1.603	2.190
G1	342	0.131	0.016	-0.008	0.036	0.161	1.629
G1	352	0.105	0.131	0.164	0.336	1.751	1.975
G2	5	-0.008	-0.008	1.010	0.791	-0.095	1.718
G2	6	0.046	0.166	0.428	0.228	1.740	1.989
G2	21	0.133	0.056	1.595	1.907	1.905	1.855
G2	23	0.084	0.168	-0.035	0.100	0.953	1.593
G2	29	0.282	0.208	0.675	0.330	1.713	2.010
G2	40	0.160	0.130	0.990	2.098	2.203	2.168
G2	59	-0.013	0.008	1.575	2.017	2.057	2.203
G2	61	0.055	0.010	0.770	0.977	0.480	0.858
G2	63	-0.030	-0.002	1.473	0.923	2.065	1.980
G2	73	0.327	0.123	1.192	1.052	1.202	2.102
G2	76	-0.038	-0.040	1.600	1.303	0.737	1.943
G2	77	0.192	0.058	0.700	0.790	NA	NA
G3	183	-0.005	0.100	0.227	0.137	0.125	2.182
G3	184	-0.043	0.093	0.278	0.302	1.875	2.235
G3	188	-0.045	-0.002	0.005	0.190	0.715	2.088
G3	190	-0.015	-0.033	-0.022	1.968	2.007	1.902
G3	191	0.413	0.333	0.050	-0.005	0.478	1.465
G3	204	0.257	0.055	0.200	0.693	1.847	2.320
G3	209	0.090	-0.037	0.012	0.238	1.505	1.683
G3	222	0.032	0.008	0.290	0.156	0.390	0.994
G3	228	0.049	-0.006	0.456	0.537	0.473	1.476
G3	239	-0.047	0.379	0.394	0.283	1.494	2.025
G3	257	-0.005	0.171	0.091	0.108	1.324	2.021
G3	262	-0.043	0.045	0.220	1.752	1.616	-0.069
G4	98	0.075	0.019	1.264	1.664	1.979	1.894
G4	99	-0.053	-0.041	1.341	1.383	1.925	2.025
G4	100	-0.036	0.135	0.108	0.148	1.551	1.883
G4	112	0.005	0.032	-0.019	0.287	0.827	0.736
G4	114	-0.018	-0.030	0.880	0.648	1.424	1.159
G4	122	0.698	0.270	0.751	0.320	1.354	1.708
G4	129	0.018	0.099	1.162	0.773	1.940	2.071
G4	131	0.006	0.034	0.025	0.194	1.098	1.092
G4	146	0.006	0.041	0.228	1.083	1.306	1.247
G4	171	1.098	1.092	0.006	0.041	0.228	1.083
G4	177	1.306	1.247	1.496	1.331	1.904	1.796
G4	179	-0.022	-0.052	-0.052	0.253	1.132	0.761
